# What are the significant factors affecting pain in patients with Hartofilakidis type Ι developmental dysplasia of the hip?

**DOI:** 10.1186/s13018-021-02761-3

**Published:** 2021-10-18

**Authors:** Yange Gu, Wenshu Jin, Han Zhang, Zhiwei Shi, Yaohui Yue, Zhaolong Yan, Zhang Zhao, Shufeng Li, Xinfeng Yan

**Affiliations:** 1 Cheeloo College of Medicine, Shandong University, 44 Wenhua West Road, Jinan, 250014 Shandong China; 2 School of Sports Medicine and Rehabilitation, Shandong First Medical University & Shandong Academy of Medical Sciences, 619 Great Wall Road, Tai’an, 271000 Shandong China; 3 Department of Orthopedic Surgery, Shandong Key Laboratory of Rheumatic Disease and Translational Medicine, The First Affiliated Hospital of Shandong First Medical University & Shandong Provincial Qianfoshan Hospital, 16766 Jing Shi Road, Jinan, 250014 Shandong China

**Keywords:** Developmental dysplasia of the hip, Acetabular dysplasia, Age at onset of pain, Severe pain

## Abstract

**Objective:**

To explore the influencing factors of age at onset of pain and severe pain in patients with Hartofilakidis type I developmental dysplasia of the hip (DDH).

**Methods:**

A retrospective study of 83 patients with DDH treated at our hospital from January 2017 to June 2021 was conducted. The age at onset of pain, patients’ demographic data, and radiographic parameters were collected. Multiple linear regression was used to determine the influencing factors of age at onset of pain. Cox regression analysis was used to determine the influencing factors of severe pain attacks.

**Results:**

According to the results of multiple linear regression analysis, when the distance between the medial femoral head and the ilioischial line increased by one millimetre, the age at onset of pain decreased by 1.7 years (*β* = − 1.738, 95% CI − 1.914–[− 1.561], *p* < 0.001). When the sharp angle increases by one degree, the age at onset of pain decreases by 0.3 years (*β* = − 0.334, 95% CI − 0.496–[− 0.171], *p* < 0.001). According to the results of the Cox regression analysis, for each additional degree of the lateral centre-edge angle (LCEA), the probability of severe pain was reduced by 5% (Exp [*β*]: = 0.947, 95% CI 0.898–0.999, *p* = 0.044). For each additional millimetre in the distance between the medial femoral head and the ilioischial line, the likelihood of severe pain increased by 2.4 times (Exp [*β*]: 2.417, 95% CI 1.653–3.533, *p* < 0.001).

**Conclusion:**

Larger distances between the medial femoral head and the ilioischial line and sharp angle can lead to an earlier age at onset of pain in patients with DDH. Small LCEA and excessive distance between the medial femoral head and the ilioischial line are risk factors for severe pain.

## Introduction

Developmental dysplasia of the hip (DDH) is characterised by insufficient coverage of the femoral head and the upper lateral inclination of the acetabular articular surface, resulting in a reduction in the contact area between the femoral head and the acetabulum, joint instability, and acetabular edge overloading, resulting in acetabular labrum and cartilage injury, eventually progressing to secondary osteoarthritis [1–3]. Many patients with DDH with symptoms and limited functions show definite pain symptoms before they develop advanced secondary osteoarthritis [4], but the reason for the difference in age and degree of painful attacks is not clear [5, 6]. Despite the growing development of treatment for patients with DDH, the optimal selection criteria for surgery and factors for symptomatic pain are still evolving. In this study, the clinical data of patients with DDH were analysed retrospectively, and the factors affecting the age at onset of pain and severe pain were explored.

## Materials and methods

### Inclusion and exclusion criteria

The inclusion criteria were patients with Hartofilakidis type I DDH treated at our hospital from January 2017 to June 2021. The exclusion criteria were ① a history of hip joint trauma; ② existing nerve, muscle, or connective tissue disease; ③ a history of hip surgery; ④ severe joint deformity; ⑤ lack of follow-up or incomplete radiographic data; ⑥ patients who could not accurately determine the age of pain onset.

### Patient data

During this period, a total of 104 patients with Hartofilakidis type I DDH were treated at our hospital. Four cases were excluded for lack of follow-up, five cases had incomplete radiographic data, and 12 patients could not accurately determine the age of pain onset. Finally, 83 patients were included in this study. The general information of the patients is shown in Table 1.

**Table 1:** General data parameters of patients

Demographic parameters	Value
Number of patients (hip)	83 (83)
pain onset age [$\overline{X} \pm S$, years]	28.3 ± 8.4
Gender [case (%)]	
Male	15 (18.1%)
Female	68 (81.9%)
Side [case (%)]	
Left	33 (39.8%)
Right	50 (60.2%)
BMI ($\overline{X} \pm S$, kg/m^2^)	23.1 ± 4.0
LCEA ($\overline{X} \pm S$, °)	9.2 ± 9.0
Sharp angle ($\overline{X} \pm S$, °)	47.9 ± 4.6
EI ($\overline{X} \pm S$)	35.7% ± 11.7%
Tonnis grade	
0 grade	11 (33.7%)
1 grade	54 (65.1%)
2 grade	1 (1.2%)
3 grade	-
Tonnis angle ($\overline{X} \pm S$, °)	20.3 ± 7.6
p/a ratio [M(IQR)]	2.2 (0.8)
AAA [M(IQR), °]	20.7 (7.8)
Shenton line [case (%)]	
Continuous	55 (66.3%)
Discontinuous	28 (33.7%)
Joint congruency [case (%)]	
Excellent	24 (28.9%)
Good	44 (53.0%)
General	14 (16.9%)
Bad	1 (1.2%)
sphericity index of femoral head [$\overline{X} \pm S$]	81.5% ± 7.0%
*d* [M(IQR), mm]	10.6 (4.5)

BMI = body mass index; *d* = distance between medial femoral head and ilioischial line

LCEA = lateral central edge angle; EI = femoral head extrusion index; AAA = acetabular anteversion angle

### Radiographic measurement and follow-up

**Fig. 1:**
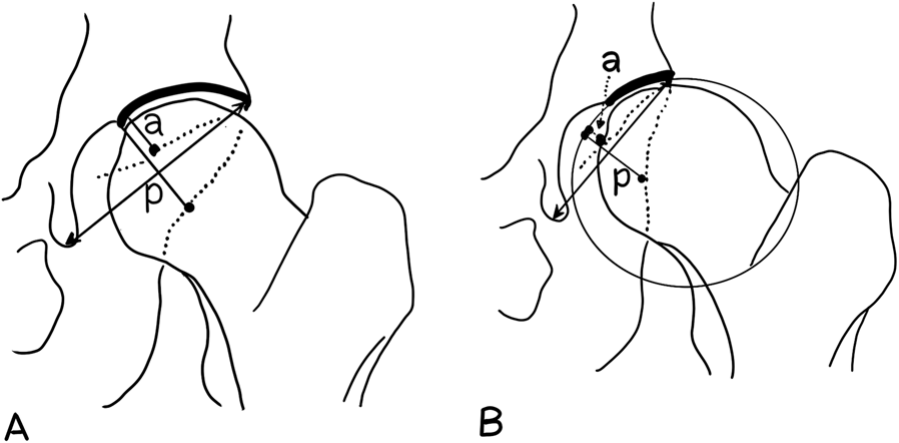
**A** The *p*/*a* ratio is calculated by dividing *p* (the distance from the acetabular joint surface to the edge of the posterior wall of the acetabulum) by *a* (the distance from the acetabular joint surface to the edge of the anterior wall of the acetabulum), and both are measured on the vertical bisector of the line connecting the teardrop and the outer edge of the acetabulum. **B** When the vertical bisector is located in the acetabular fossa, the acetabular fossa is ignored. The acetabular articular surface is used as a part of the circle to make the best-fit circle, and the intersection of the trajectory of the circle and the vertical bisector is taken as the point of p and a near-point

**Fig. 2:**
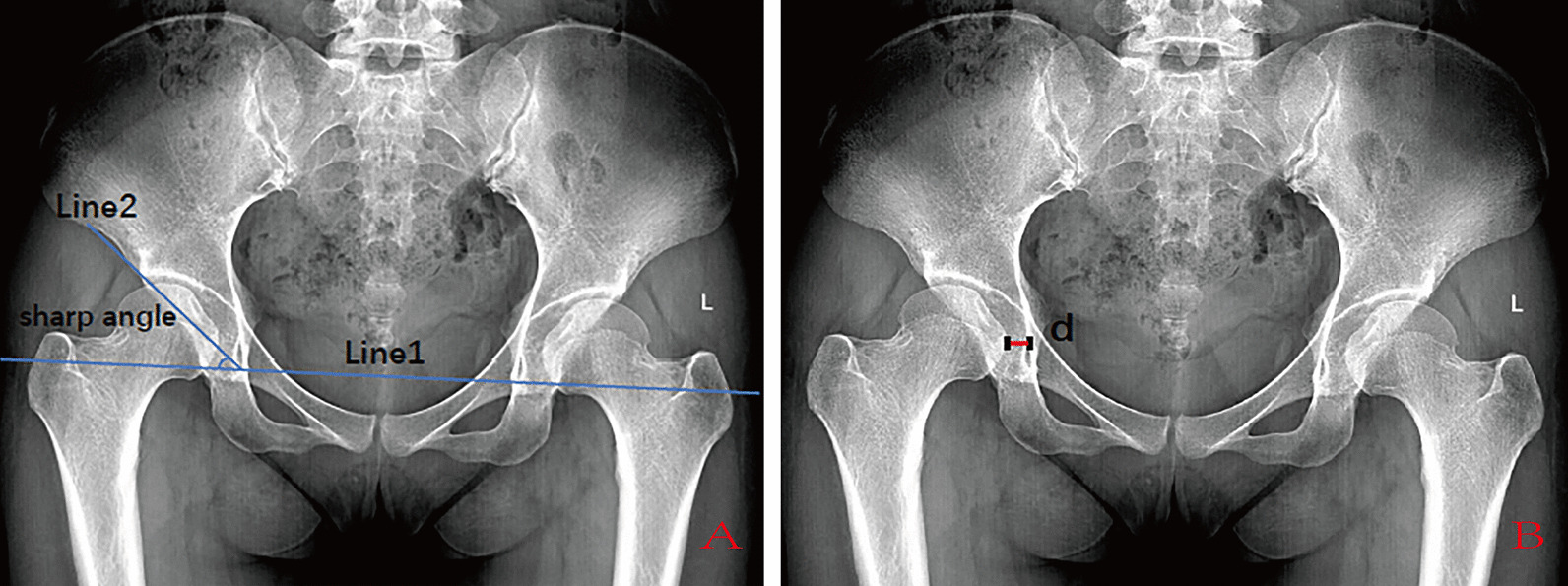
**A** Line 1 is the teardrop connection. Line 2 is the line connecting the teardrop and the outer edge of the acetabulum. The angle between the two lines is the sharp angle. **B** *d* is the distance between the medial femoral head and the ilioischial line

A well-trained observer completed the measurement process independently in anterior and posterior x-rays of both hips. Radiographic parameters included the acetabular top tilt angle (Tonnis angle), lateral centre-edge angle (LCEA), sharp angle, femoral head extrusion index (EI), sphericity index of the femoral head [7], Shenton line, osteoarthritis Tonnis grade [8], joint congruency [9], P/A ratio and corresponding acetabular anteversion angle (AAA) [10], and the distance between the medial femoral head and the ilioischial line [11]. The patients’ age at onset of pain [5, 12] and Western Ontario and McMaster Universities Osteoarthritis Index (WOMAC) pain scores were collected. The age at which any item of persistent pain described in the WOMAC pain score scale occurs for the first time is the age at onset of pain [5, 9, 12]. A WOMAC pain score ≥ 10 was taken as the criterion of severe pain attack [9]. The method of measuring the *P*/*A* ratio [10] is shown in Fig. 1. The method of measuring the sharp angle [13] and the distance between the medial femoral head and the ilioischial line [11] is shown in Fig. 2.

### Statistical methods

A Kolmogorov–Smirnov test was used to test the normal distribution of quantitative variables. Normally distributed measurement data were expressed as mean ± standard deviation ($\overline{X }\pm S$). The skew distribution measurement data were represented as median (interquartile range) (*M*[IQR]). Categorical variables were expressed as a percentage (%). The patients’ general demographic data and radiographic parameters were analysed by a single-factor linear regression analysis taking the age at onset of pain as the dependent variable, and the test level *α* was set as 0.05. If the independent variable was *p* < 0.05 in a single-factor linear regression analysis, the variable was included in the multiple linear regression model. According to the statistical results determining which variable influences the age at onset of pain factor, *p* < 0.05 for the difference was statistically significant. The age at onset of pain was taken as the time variable and the WOMAC pain score ≥ 10 as the state variable. The demographic data and radiographic parameters of the patients were analysed by a single-factor Cox regression analysis. If the independent variable was *p* < 0.05 in the results of a single-factor Cox regression analysis, the variable was included in the Cox proportional hazard model, and which variables were the influencing factors of severe pain were determined according to the statistical results. All the statistical analyses were performed with SPSS software for Windows (version 25.0, SPSS, New York, NY, USA).

## Results

### Factors affecting the age at onset of pain

The demographic data of patients with pain (sex, body mass index, side) and the various radiographic parameters (Tonnis angle, LCEA, sharp angle, EI, sphericity index of the femoral head, Shenton line, osteoarthritis Tonnis grade, joint congruency, P/A ratio and corresponding AAA, and the distance between the medial femoral head and the ilioischial line) were screened out by a single-factor linear regression analysis of the distance between the medial femoral head and the ilioischial line (*p* < 0.001) and the sharp angle (*p* = 0.012) (Table 2).

**Table 2:** Results of single factor linear regression analysis

Variables	*β*	*βeta*	*t*	95%*CI*	*p*
Sex	− 1.928	− 0.089	− 0.806	− 6.689–2.832	0.423
BMI	0.439	0.205	1.884	− 0.025–0.903	0.063
Side	0.922	0.054	0.489	− 2.830–4.675	0.626
Sphericity index	− 0.249	− 0.208	− 1.913	− 0.509–0.010	0.059
AAA	0.027	0.028	0.254	− 0.183–0.237	0.800
LCEA	0.033	0.035	0.315	− 0.174–0.240	0.753
Sharp angle	− 0.501	− 0.275	− 2.579	− 0.888– (− 0.114)	0.012^*^
EI	− 0.033	− 0.047	− 0.420	− 0.192–0.125	0.675
Shenton line	− 0.042	− 0.002	− 0.021	− 3.931–3.848	0.983
Tonnis grade	2.496	0.148	1.347	− 1.189–6.181	0.182
Joint congruency	1.007	0.085	0.770	− 1.593–3.607	0.443
Tonnis angle	0.016	0.014	0.128	− 0.227–0.258	0.899
*d*	− 1.776	− 0.899	− 18.429	− 1.967–(− 1.584)	< 0.001^*^

BMI = body mass index; AAA = acetabular anteversion angle; LCEA = lateral central edge angle; EI = femoral head extrusion index; *d* = distance between medial femoral head and ilioischial line

*p < 0.05

The distance between the medial femoral head and the ilioischial line and sharp angle was included in multiple linear regression. It was found that there was significant statistical significance between them and age at onset of pain (Table 3). According to the results of multiple linear regression analysis, when the distance between the medial femoral head and the ilioischial line increased by one millimetre, the age at onset of pain decreased by 1.7 years (*β* = − 1.738, 95% CI − 1.914–[− 1.561], *p* < 0.001). When the sharp angle increases by one degree, the age at onset of pain decreases by 0.3 years (*β* = − 0.334, 95% CI − 0.496–[− 0.171], *p* < 0.001). No statistical correlation was found between other demographic data or radiographic parameters and the age of pain onset.

**Table 3:** Results of multiple linear regression

Variables	*β*	*βeta*	*t*	95%*CI*	*p*
Sharp angle	− 0.334	− 0.183	− 4.084	− 0.496– (− 0.171)	< 0.001
*d*	− 1.738	− 0.879	− 19.595	− 1.914– (− 1.561)	< 0.001

*d* = distance between medial femoral head and ilioischial line

### Influencing factors of severe pain

Cox regression analysis was used to screen the demographic data of the patients (sex, body mass index, side) and the various radiographic parameters listed above. The corresponding independent variables screened out were the distance between the medial femoral head and the ilioischial line (*p* < 0.001), LCEA (*p* = 0.045), and the sharp angle (*p* = 0.036) (Table 4). The selected independent variables were included in the Cox regression analysis, and the results showed that for each additional degree of the LCEA, the probability of severe pain reduced by 5% (Exp (*β*) = 0.947, 95% CI 0.898–0.999, *p* = 0.044). For each additional millimetre in the distance between the medial femoral head and the ilioischial line, the likelihood of severe pain increased by 2.4 times (Exp (*β*): 2.417, 95% CI 1.653–3.533, *p* < 0.001). An omnibus test showed that the constructed Cox regression model was statistically significant (− 2 loglike: 128.132, *p* < 0.001). No statistical correlation was found between other demographic data or radiographic parameters and severe pain.

**Table 4:** Results of single factor COX regression analysis

Variables	*β*	*wald*	*Exp (β)*	95%*CI*	*p*
Sex	− 0.064	0.014	0.938	0.323–2.727	0.907
BMI	0.059	1.390	1.061	0.962–1.171	0.238
Side	0.289	0.458	1.334	0.579–3.077	0.498
Sphericity index	0.057	3.608	1.059	0.998–1.124	0.058
AAA	0.012	0.471	1.012	0.979–1.046	0.492
LCEA	− 0.041	4.031	0.960	0.922–0.999	0.045*
Sharp angle	0.104	4.410	1.110	1.007–1.224	0.036*
EI	0.015	0.741	1.015	0.981–1.052	0.389
Shenton line	0.243	0.361	1.275	0.578–2.814	0.548
Tonnis grade	0.315	0.498	1.371	0.571–3.291	0.480
Joint congruency	− 0.172	0.379	0.842	0.486–1.457	0.538
Tonnis angle	0.046	3.456	1.047	0.998–1.099	0.063
*d*	0.816	18.029	2.262	1.552–3.298	< 0.001*

BMI = body mass index; AAA = acetabular anteversion angle; LCEA = lateral central edge angle; EI = femoral head extrusion index; *d* = distance between medial femoral head and ilioischial line

*p < 0.05

## Discussion

### Main findings

The clinical symptoms of patients with DDH are primarily related to the increase in local mechanical stress and dynamic hip instability caused by insufficient coverage of the hip joint [14]. Although reports on the causes of pain symptoms are gradually increasing, the influencing factors of age at onset of pain and pain degree are still not completely clear [6, 14].

In this study, we found that a large distance between the medial femoral head and the ilioischial line and the sharp angle can lead to an earlier age at onset of pain in patients with DDH; a small LCEA and a large distance between the medial femoral head and the ilioischial line are the risk factors for severe pain (Table 5).

**Table 5:** Results of COX regression analysis

Variables	*β*	wald	Exp (*β*)	95%CI	*p*
LCEA	− 0.054	4.057	0.947	0.898–0.999	0.044*
*d*	0.883	20.753	2.417	1.653–3.533	< 0.001*
Sharp angle	0.076	1.731	1.078	0.964–1.207	0.188

LCEA = lateral central edge angle; *d* = distance between medial femoral head and ilioischial line

*p < 0.05

### Influencing factors of age at onset of pain

The external movement of the centre of the hip joint is one of the imaging findings of patients with DDH. John C. Clohisy uses the distance between the medial femoral head and the ilioischial line on x-rays to describe the degree of external movement of the centre of the hip joint and proposes that the distance between the medial femoral head and the ilioischial line is 0–10 mm [11]. The external movement of the centre of the hip joint in patients with DDH lengthens the gravity lever arm and increases the joint reaction [15], which may lead to earlier onset of hip pain and increase the likelihood of severe pain. Sharp angle reflects acetabular development and its coverage of the femoral head and can be used to diagnose and predict the progress of DDH. The normal reference value [16] is 38°–42°.

We found that the sharp angle negatively affects the age at onset of pain of patients with DDH, which may be due to the increase in joint contact pressure caused by insufficient coverage of the femoral head when the sharp angle is large. The subsequent static overload leads to the degeneration of articular cartilage, and the overload of soft tissue structure is the ultimate common cause of pain in patients with DDH [6].

Yusuke Kohno et al. also found that an extremely sharp angle is associated with early pain in patients with dysplastic hips [12]. In addition, they also suggested that combined anteversion is a risk factor for the early onset of pain. The combined anteversion is the sum of the femoral anteversion angle and the AAA, which represents the morphological relationship between the two on the axial position. The increase in the femoral anteversion angle and the shortening of the femoral neck led to the early development of secondary osteoarthritis [17]. We speculate that the AAA may also be one of the independent factors affecting the age at onset of pain. We considered this factor and conducted a single-factor analysis, but it was not statistically significant.

Previous studies have shown that acetabular retroversion is related to the decrease in the coverage area of the femoral head, and hip pain occurs earlier in patients with acetabular dysplasia with acetabular retroversion than those without acetabular retroversion [18]. Since there was no acetabular retroversion in the x-rays of 83 patients, we did not verify it.

### Small LCEA is a risk factor for severe pain

Small LCEA is a risk factor for severe pain in patients with DDH, which is related to the contact area of the femoral head and the biomechanical state of the hip joint. LCEA is an independent factor affecting the contact area of the femoral head. The smaller LCEA limits the area that can be used for proper load distribution and increases the hip joint instability, which leads to articular cartilage injury [19]. In patients with DDH, the contact stress of the hip joint increases sharply to the lateral edge, while the increased LCEA can improve the lateral coverage of the femoral head, reduce the contact stress of the hip joint and change the position of the peak contact stress [20, 21]. A hip joint with a larger LCEA has a larger contact area of the femoral head and a better biomechanical state, which is the main reason why severe pain does not occur easily.

In addition, Eduardo N. Novais et al. [20] found that preoperative LCEA is an independent influencing factor of LCEA < 22° after a Bernese periacetabular osteotomy, and patients with DDH with lower preoperative LCEA are more likely to have an inadequate correction, resulting in surgical failure. Therefore, for patients with small LCEA, how to choose the appropriate operation time and achieve an effective correction of LCEA still needs to be further studied.

### Limitations of the study

This study had certain limitations, however. First, our sample size was small. Second, our study was a retrospective case study. This type of study inherently has various sources of bias, including selection bias, measurement and evaluation bias, as well as lack of follow-up. Finally, this was a short-term follow-up study, and it did not provide medium- to long-term follow-up results.

## Conclusions

A larger distance between the medial femoral head and the ilioischial line and sharp angle can lead to earlier age at onset of pain in patients with DDH. A small LCEA and excessive distance between the medial femoral head and the ilioischial line are risk factors for severe pain. How to define the surgical indication more accurately, choose the best operation time, and realise the effective correction of hip joint deformity still needs further research.

## Data Availability

The data are not publicly available, as participants of this study did not agree for their data to be shared publicly.

## Abbreviations

DDH: Developmental dysplasia of the hip
LCEA: Lateral centre-edge angle
EI: Extrusion index
AAA: Acetabular anteversion angle
WOMAC: Western Ontario and McMaster Universities Osteoarthritis Index
